# Phonemic restoration in developmental dyslexia

**DOI:** 10.3389/fnins.2014.00134

**Published:** 2014-06-04

**Authors:** Stephanie N. Del Tufo, Emily B. Myers

**Affiliations:** ^1^Department of Psychology, University of ConnecticutStorrs, CT, USA; ^2^Haskins LaboratoriesNew Haven, CT, USA; ^3^Department of Speech, Language, and Hearing Sciences, University of ConnecticutStorrs, CT, USA

**Keywords:** dyslexia, phonemic restoration, specific reading disability, speech perception, phonological awareness, phonological processing, categorical perception, phonetics

## Abstract

The comprehension of fluent speech in one's native language requires that listeners integrate the detailed acoustic-phonetic information available in the sound signal with linguistic knowledge. This interplay is especially apparent in the phoneme restoration effect, a phenomenon in which a missing phoneme is “restored” via the influence of top-down information from the lexicon and through bottom-up acoustic processing. Developmental dyslexia is a disorder characterized by an inability to read at the level of one's peers without any clear failure due to environmental influences. In the current study we utilized the phonemic restoration illusion paradigm to examine individual differences in phonemic restoration across a range of reading ability, from very good to dyslexic readers. Results demonstrate that restoration occurs less in those who have high scores on measures of phonological processing. Based on these results, we suggest that the processing or representation of acoustic detail may not be as reliable in poor and dyslexic readers, with the result that lexical information is more likely to override acoustic properties of the stimuli. This pattern of increased restoration could result from a failure of perceptual tuning, in which unstable representations of speech sounds result in the acceptance of non-speech sounds as speech. An additional or alternative theory is that degraded or impaired phonological processing at the speech sound level may reflect architecture that is overly plastic and consequently fails to stabilize appropriately for speech sound representations. Therefore, the inability to separate speech and noise may result as a deficit in separating noise from the acoustic signal.

## Introduction

Developmental dyslexia refers to an inability to read at grade level despite adequate instruction, intellectual ability, motivation and regardless of socioeconomic status (Berninger, 2001). Developmental dyslexia, which will be referred to as dyslexia for the remainder of this paper, is estimated to effect 4–12% of the population and is resilient into adulthood (see Gabrieli, 2009 for review). Deficits in phonological awareness have long been the primary hallmark of dyslexia (Bradley and Bryant, 1978; Liberman et al., 1989). An effort to discover a more general deficit, that culminates in spectrum of reading difficulty, has led to this population being described as having a specific reading disability (RD) (see Shaywitz and Shaywitz, 2005 for review). It is hotly debated whether this reading impairment arises from deficits in phonological awareness, rapid auditory processing, visual motion, or noise exclusion. It has been suggested that the phonological deficit in those with dyslexia may originate from an auditory-perceptual deficit (Richardson et al., 2004). This hypothesis stems from evidence of impaired talker identification (Perrachione et al., 2011), frequency discrimination (Banai and Ahissar, 2004; Ahissar et al., 2006; Ahissar, 2007) and difficulty with amplitude modulation (Goswami et al., 2002; Richardson et al., 2004). There has also been some evidence suggesting that individuals with dyslexia exhibit a more general speech perception deficit (Manis et al., 1997; Hazan, 1998).

In studies of speech perception in quiet, only a small portion of dyslexic individuals struggled with speech perception (Manis et al., 1997; Adlard and Hazan, 1998). Evidence from Ziegler et al. (2009) suggests that dyslexic children struggle with speech perception in noise more profoundly than their age-matched or reading level-matched peers. This suggests that speech perception in dyslexia may be more susceptible to noise interference than in age-matched or reading-matched peers (Ziegler et al., 2009). This result is very similar to the speech-in-noise deficit seen in children with a specific language impairment (SLI) (Ziegler et al., 2005). Furthermore, in speech-in-noise paradigms, children with language learning disabilities, have shown difficulty with speech-in-noise paradigms behaviorally (Bradlow et al., 2003), electrophysiologically at the level of brainstem (Cunningham et al., 2001; Russo et al., 2005) and in the cortex (Warrier et al., 2004; Wible et al., 2005). Phonological processing deficits have been shown in both populations with SLI (Fidler et al., 2011) and in those with language learning disabilities (Richman, 1983). Taken together, this suggests that speech-in-noise perceptual differences do exist between individuals with different reading ability.

Speech-in-noise perceptual differences in dyslexia could be occurring due to a failure in noise exclusion or a more general lack of speech restoration strength. “Failure in auditory noise exclusion” refers to the hypothesis that speech and noise are weighted the same to the dyslexic auditory system and that speech has no special value (Sperling et al., 2005). An alternative hypothesis is that individuals lack sufficient “phonemic restoration strength.” By this we mean that individuals with dyslexia may process speech normally, but a lack of robustness in the dyslexic auditory system may lead to perceptual degradation in the speech signal. This would lead to difficulties in filling in gaps in the acoustic signal when portions of that signal are occluded by noise. In the current investigation, we ask whether individual differences in reading skill are related to differences in phoneme restoration.

Phonemic restoration is an auditory illusion that requires the integration of bottom-up information from the acoustic signal, seamlessly coordinated with top-down lexical status expectations generated by the listener's prior knowledge. Warren (1970) first demonstrated this phenomenon when he showed that if a sound, such as a cough or tone, replaces a speech sound, listeners believe they hear the missing sound or phoneme. This illusory percept of the missing phoneme is referred to as phoneme restoration. However, if silence replaces a speech sound, the interval is detected, the listener notes the interruption of the word and phonemic restoration does not occur; this is known as a failure to restore (Warren, 1970; Warren and Obusek, 1971; Repp, 1991). The phonemic restoration experimental paradigm allows unique insight into the perceptual mechanism of verbal word recognition.

Evidence that phonemic restoration relies on bottom-up information from the acoustic signal comes from the initial phonemic restoration experiment where Warren (1970) demonstrated that a failure to restore occurred if silence replaced a speech sound, suggesting that some acoustic signal must fill the gap in order for an illusory phoneme to be restored. The strength of restoration effects are conditioned by the nature of the occluding stimulus. In particular, evidence from Bashford et al. (1996) has shown if the replacement non-speech sound has a matching amplitude envelope to the sound replaced, restoration increases. Increased restoration through psychoacoustic correspondence of the replacement sound to the sound replaced suggests that perhaps bottom up evidence is strengthened due to mechanisms in the speech perception system normally employed to correct for errors in speech (Frisch and Wright, 2002) or possibly utilized in acoustic variability across speakers (Warren and Obusek, 1971; Layton, 1975; Bashford and Warren, 1979, 1987; Samuel, 1981b; Verschuure and Brocaar, 1983; Bashford et al., 1992). Taken together, these studies provide clear evidence that bottom-up acoustics of the speech signal play a significant role in phonemic restoration.

Evidence for the effects of top-down information on phoneme restoration comes primarily from paradigms investigating words and pseudowords. Restoration is thought to be guided by top-down influences from the listener's lexical knowledge (Samuel, 1987). Restoration occurs more quickly and frequently in tokens with a lexical status (e.g., words) (Samuel, 1981a, 1996). This suggests that lexical knowledge increases the strength of the illusory phoneme. Lexical effects were further tested through word length, with longer words increasing restoration (Samuel, 1981a, 1996; Bashford et al., 1988). Additional evidence suggests that restoration is stronger for phonemes that occur later in words (Marslen-Wilson and Welsh, 1978). This has been posited to be due to the strong expectations regarding the identity of the missing phoneme facilitate restoration.

The phoneme restoration paradigm allows for the examination of both top-down and bottom-up aspects of speech-in-noise perception. Utilizing this paradigm in dyslexia will provide unique insight into a possible speech-in-noise deficit in dyslexia. Prior evidence suggests that phonemic restoration requires that the listener have both intact bottom-up acoustic processing as well as top-down information from the lexicon to perform restoration for real words. At a pseudoword condition level, lexical information is available from the lexical neighborhood that is, activation of the lexical neighborhood may provide enough information to allow the listener to restore the missing phoneme (Samuel, 1987); however, pseudowords do not have a lexical entry. The lexical information available for real words will be stronger and more specific than for pseudowords (Samuel, 1996). Pseudoword phoneme restoration requires intact (bottom-up) processing of the acoustic details of the speech signal as well as access to a more abstract top-down representation of the lexical neighborhood. In the context of isolated speech sounds, that is, speech sounds excised from words, listeners are required to have an intact acoustic representation of a speech sound, in this case the /s/ fricative. Prior results from Samuel (1981a) with an ecologically valid sample demonstrate that phonemic restoration effects are strongest in words compared to pseudowords, and weakest with individual speech sound segments that have been excised from words. This gradation in the strength of the restoration effect presumably results from the variable degree of top-down information that is available in words vs. pseudowords vs. speech sound segments.

Traditional speech paradigms have reported mixed results in individuals with dyslexia. Children with dyslexia show a deficit in speech perception, specifically in noise (Ziegler et al., 2009). Prior work has shown that only a small portion of dyslexic individuals show degraded speech perception (Manis et al., 1997; Adlard and Hazan, 1998). One advantage of the phonemic restoration paradigm is that it does not rely on a failure to perform the task as evidence to support a hypothesis. In essence, the typical pattern of restoration does reflect a “failure” to detect the absence of the speech sound. As such, we aim to determine whether performance on restoration tasks is similar across participants on a spectrum of good-to-dyslexic readers.

Here we aim to address the relationship between reading ability and phonemic restoration. One possibility is that better readers will be better at detecting the interruption in speech (restoration will not occur). This outcome would suggest that good readers are not fooled by the illusion of restoration and instead have very clear representations of individual speech sounds, that is, their bottom-up processing is high fidelity. An alternative possibility is that better readers will be worse at detecting the interruption in speech (restoration will occur). This would suggest that good readers are better able to adapt to deviations in the pronunciation of speech sounds, possibly in order to handle individual variability. It is furthermore possible that a specific aspect of reading ability, such as phonological processing or comprehension, is more closely tied to phonemic restoration than measures of overall reading ability.

## Materials and methods

### Participants

College students (*n* = 53; male = 18) were recruited from the University of Connecticut and screened with approval from the Office of Research Compliance. Participants received either extra course credits or payment for participation. Participants were of typical college age (Mean Age = 19.74, Standard Error (SE) = 0.31), were right-handed based on questionnaire responses adapted from the *Edinburgh Handedness Inventory*; (Oldfield, 1971), and were monolingual native speakers of American English. According to self-report, these participants had no neuropsychological conditions, less than 1 year of musical instruction, normal hearing, and full term births. Participants had no immediate family members with diagnosed developmental disorders and were taking no prescribed medication other than birth control at the time of study participation.

### Standardized behavioral testing

Given that previous studies have suggested that dyslexic populations vary on degree of impairment (Torgesen, 2005), we attempted to collect phonemic restoration data across a range of reading ability and disability (see Table 1). Standardized measures of cognitive, reading and reading-associated abilities were administered. Participants were assessed for cognitive ability based on performance intelligence quotient from the *Wechsler Abbreviated Scale of Intelligence, 3rd Ed.*, WASI-3; (Wechsler, 1999) as well as working memory from the *Wechsler Adult Intelligence Scale*, WAIS-IV; (Wechsler, 2008). Participants were also administered a reading battery including reading comprehension (“Passage Comprehension”; *Woodcock Reading Mastery Tests-Third Edition*, WRMT-III; (Woodcock, 2011) and phonological processing “Elision,” “Blending Words,” and “Non-word Repetition”; *Comprehensive Test of Phonological Processing*, CTOPP; (Wagner et al., 1999). Participants were administered standardized assessments of timed “Sight Word Efficiency” and “Phonemic Decoding Efficiency”; *Test of Word Reading Efficiency*, TOWRE; (Torgesen et al., 1999) and untimed measures “Word Identification” and “Word Attack”; *(Woodcock Reading Mastery Tests-Third Edition, WRMT-III)* of single word as well as pseudoword reading. A standardized assessment of timed sentence reading, in which a semantic judgment was made, were used to assess reading fluency “Reading Fluency”; *Woodcock Johnson III*, WJIII; (Woodcock et al., 2007).

**Table 1:** **Behavioral assessment scores**.

**TEST subtest**	**Raw scores**		**Standardized scores**		***d***′ **words correlation**		***d***′ **speech sound segments correlation**	
	**Score**	**Range**	**Score**	**Range**	***r***	***p*-value**	***r***	***p*-value**
**WASI**								
Block design	52.65 ± 1.26	(35–70)	55.81 ± 0.85	(45–67)	–	–	–	–
Matrix reasoning	28.0 ± 0.34	(23–32)	53.52 ± 0.72	(43–64)	–	–	–	–
Performance IQ	109.54 ± 1.19	(90–126)	107.19 ± 1.08	(92–123)	–	–	–	–
**CTOPP**								
Elision	17.98 ± 0.19	(14–20)	10.08 ± 0.20	(7–13)	0.25	0.07	0.16	0.25
Blending words	17.15 ± 0.50	(7–20)	11.43 ± 0.41	(4–15)	0.3	0.03^*^	0.3	0.03^*^
Non-word repetition	11.40 ± 0.38	(6–18)	8.45 ± 0.43	(4–25)	0.35	0.009^**^	0.05	0.74
**WRMT-III**								
Word ID	40.49 ± 0.36	(31–45)	99.21 ± 1.34	(68–118)	−0.04	0.73	0.09	0.53
Word attack	21.79 ± 0.31	(16–26)	93.72 ± 1.63	(68–121)	0.18	0.18	0.21	0.13
Passage comprehension	32.19 ± 0.43	(22–38)	103.66 ± 1.54	(74–128)	0.23	0.09	0.02	0.9
**TOWRE**								
Sight word reading	93.28 ± 1.07	(76–104)	97.04 ± 1.46	(80–113)	0.12	0.38	0.05	0.74
Decoding	51.57 ± 0.85	(37–62)	96.49 ± 1.29	(81–120)	0.07	0.65	0.09	0.52
**WJIII**								
Fluency	82.11 ± 1.94	(58–136)	103.43 ± 1.69	(77–145)	0.21	0.13	−0.08	0.6
**WAIS-IV**								
Digit span total	27.70 ± 0.62	(19–36)	10.06 ± 0.36	(5–19)	–	–	–	–
**DEMOGRAPHICS**								
Age (years)	19.74 ± 0.3							
Sex	18M/35F							
Total N	*N* = 53							

*Values are mean ± SE. Range shows the minimum and maximum values*.

Asterisks indicate

* *p < 0.05*,

** *p < 0.01*.

Inclusion criteria were based on dyslexia literature (Katzir et al., 2008; Katzir, 2009) (see Table 2). Good readers (*n* = 23; male = 8) all scored above the bottom 25th percentile on all measures of timed and untimed word and pseudoword reading (Woodcock, 2011) in addition to having no self-reported history of reading difficulty. Poor readers (*n* = 14; male = 5) did not report any history of difficulty of reading but nonetheless scored below the 25th percentile on at least one measure of timed word or pseudoword reading. Dyslexic readers (*n* = 16; male = 5) self-reported either a diagnosis of dyslexia or a continuous history of reading difficulty and remediation; dyslexic readers scored below the 25th percentile on two or more measures of timed or untimed word or pseudoword reading. Importantly, relatively poor scores on measures of phonological processing may be seen even among individuals with no previous history of reading difficulty. In order to examine potential differences between individuals with a reported reading disability compared to those with no documented reading disability, participants were grouped based on standardized testing scores and self-report via questionnaire into three groups: good readers, poor readers and dyslexic readers. However, preliminary analysis (see Table 3) suggested that poor readers and dyslexic readers were performing very similarly across restoration conditions. Therefore, all group analysis shown in the result section of this paper will reflect two groups, good readers (*n* = 23; male = 8) and poor-to-dyslexic readers (*n* = 30; male = 10).

**Table 2:** **Participant behavioral assessment demographics**.

**TEST subtest**	**Good readers (GR)**		**Poor readers (PR)**		**Dyslexic readers (DR)**		**η2**	***p*-value**	**Differences^§^**
	**Raw score**	**Standard score**	**Raw score**	**Standard score**	**Raw score**	**Standard score**			
**WASI**									
Block design	54.74 ± 1.77	56.78 ± 1.23	49.86 ± 2.46	53.71 ± 1.60	52.07 ± 2.52	56.27 ± 1.71	0.05	0.32	GR = PR = DR
Matrix reasoning	27.91 ± 0.53	53.57 ± 1.11	28.07 ± 0.74	54.0 ± 1.45	27.47 ± 0.60	53.0 ± 1.29	0.005	0.87	GR = PR = DR
Performance IQ	110.35 ± 1.94	108.17 ± 1.65	107.71 ± 2.26	105.64 ± 1.94	109.06 ± 2.55	107.13 ± 2.18	0.02	0.65	GR = PR = DR
**CTOPP**									
Elision	18.43 ± 0.28	10.57 ± 0.26	18.07 ± 0.36	10.21 ± 0.41	17.25 ± 0.35	9.25 ± 0.35	0.15	**0.005**	**GR > DR**
Blending words	17.74 ± 0.65	12.04 ± 0.52	17.07 ± 0.96	11.0 ± 0.91	16.38 ± 1.09	10.94 ± 0.81	0.03	0.26	GR = PR = DR
Non-word repetition	11.91 ± 0.64	9.35 ± 0.88	11.29 ± 0.73	7.93 ± 0.58	10.75 ± 0.55	7.63 ± 0.39	0.06	0.096	GR = PR = DR
**WRMT-III**									
Word ID	41.83 ± 0.34	104.3 ± 1.49	40.43 ± 0.47	98.00 ± 1.56	38.63 ± 0.84	92.56 ± 2.88	0.28	**0.00006**	**GR > PR > DR**
Word attack	23.43 ± 0.26	104.91 ± 1.73	20.79 ± 0.45	87.79 ± 2.12	20.31 ± 0.57	86.19 ± 2.74	0.43	**0.000001**	**GR > PR >D R**
Passage comprehension	32.43 ± 0.66	104.91 ± 2.29	32.69 ± 0.71	103.05 ± 3.04	31.69 ± 0.93	102.38 ± 2.97	0.01	0.50	GR = PR = DR
**TOWRE**									
Sight word reading	97.35 ± 1.15	102.17 ± 1.97	93.14 ± 2.02	96.43 ± 2.67	87.56 ± 1.86	90.19 ± 2.26	0.23	**0.0003**	**GR > DR**
Decoding	56.30 ± 0.88	103.87 ± 1.75	51.43 ± 0.70	94.64 ± 0.85	44.88 ± 1.07	87.5 ± 1.08	0.56	**1.529^−10^**	**GR > PR > DR**
**WJIII**									
Fluency	87.57 ± 3.29	107.09 ± 2.96	83.21 ± 3.44	105.14 ± 2.77	73.31 ± 1.83	96.69 ± 2.02	0.14	**0.009**	**GR > DR**
**WAIS-IV**									
Digit span total	28.74 ± 0.93	10.61 ± 0.63	28.36 ± 1.27	10.36 ± 0.55	25.63 ± 0.99	9.0 ± 0.58	0.07	0.15	GR = PR = DR
**DEMOGRAPHICS**									
Age (years)	19.43 ± 0.39		19.64 ± 0.25		20.25 ± 0.83		0.02	0.54	GR = PR = DR
Sex	8M/15F		5M/9F		5M/11F		–	–	–
Total N	*N* = 23		*N* = 14		*N* = 16		–	–	–

*Values are mean ± SE. Effect size difference of the group is based on standard scores (η^2^)*.

§ *Differences reported (in bold) are significant at a Bonferroni α = 0.05. The general linear hypothesis was used in all individual comparisons*.

**Table 3:** **Restoration by group**.

**Group**	**Words**			**Pseudowords**			**Speech sound segments**		
	***d'***	**Beta**	**Miss**	***d'***	**Beta**	**Miss**	***d'***	**Beta**	**Miss**
Good readers	0.68 ± 0.07	0.82 ± 0.02	14.26 ± 1.50	0.99 ± 0.08	0.63 ± 0.03	24.48 ± 0.99	^§^2.47 ± 0.16	0.24 ± 0.04	20.26 ± 2.55
Poor readers	0.52 ± 0.04	0.88 ± 0.01	13.14 ± 1.86	1.16 ± 0.09	0.56 ± 0.04	29.86 ± 1.45	1.94 ± 0.21	0.39 ± 0.06	22.07 ± 3.26
Dyslexic readers	0.58 ± 0.06	0.87 ± 0.02	12.25 ± 1.59	0.96 ± 0.09	0.62 ± 0.04	30.06 ± 1.16	1.90 ± 0.17	0.40 ± 0.06	22.81 ± 2.89
Poor-to-dyslexic readers	0.56 ± 0.03	0.87 ± 0.01	12.67 ± 1.19	1.05 ± 0.06	0.59 ± 0.03	29.97 ± 0.89	^§^1.92 ± 0.13	0.39 ± 0.04	22.47 ± 2.13
All subjects	0.62 ± 0.04	0.85 ± 0.01	13.58 ± 6.79	1.02 ± 0.05	0.61 ± 0.02	27.60 ± 0.71	2.23 ± 0.11	0.31 ± 1.57	2.24 ± 0.82

*Values are mean ± SE. All within group conditions (Words, Pseudowords, Speech Sound Segments) are significant p = 3.315^−31^ based on a Post-hoc pairwise Bonferroni corrected α of 0.017. A between groups post-hoc Bonferroni comparison α of 0.017 reveled a significant effect p = 0.010 for the speech sound segment condition. Using a Bonferroni corrected α of 0.025; a pairwise group mean comparison was performed at the speech sound segment condition*.

§ *A significant difference in speech sound segment restoration p = 0.010 was found between good readers and poor-to-dyslexic readers*.

### Stimuli

Fifty three-syllable nouns with a medial /s/ fricative as the target phoneme were recorded by a female native English speaker for the full paradigm. Ten three-syllable nouns with a medial /s/ fricative as the target phoneme were recorded by a male native English speaker to be used in the practice version of the paradigm. Stimuli were normed for age of acquisition, written and oral frequency, concreteness and imageability using the MRC Psycholinguistic Database (http://websites.psychology.uwa.edu.au/school/MRCDatabase/uwa_mrc.htm). Three-syllable pseudowords with a medial /s/ fricative were created and recorded. Pseudowords were created by replacing two phonemes in each of the real words that were used for recording. Substituted phonemes were from a similar phonological class (e.g., vowels were replaced with vowels).

Recordings were altered using Pratt (http://www.fon.hum.uva.nl/praat/). Two versions of each stimulus were created: “Replaced” and “Added” stimuli. For both types of stimuli, the boundaries of the /s/ was located within the waveforms. These /s/ segments varied in duration between 100–140 ms long. Next, white noise was created in Pratt using the Random Gauss function. The formula used was randomGauss (0, 0.25) with the amplitude and duration of white noise matched individually to each stimulus's /s/ fricative. “Replaced” stimuli were created by entirely replacing the /s/ with amplitude- and duration-matched white noise. Thus, the amplitude and duration of the medial segment of the “Replaced” stimuli (e.g., white noise) was matched to the amplitude and duration of the previous /s/ fricative in each individual stimuli. “Replaced” stimuli are stimuli in which the word is missing the medial /s/ and hearing white noise (e.g., the stimulus is interrupted). “Added” stimuli were created by blending (averaging together) the /s/ segment with its amplitude and duration matched white noise, using waveform averaging in Pratt, then inserting this blend back into the word or pseudoword in the medial /s/'s previous location. Thus, the amplitude and duration of the medial segment of the “Added” stimuli was a function of the amplitude and duration of the previous /s/ fricative in each individual stimuli. “Added” stimuli are stimuli in which the word is intact but the medial /s/ includes white noise (e.g., the stimulus is intact, with added white noise). The speech sound segment condition consisted of either one of the white noise segments created for insertion into the “Replaced” condition; or one of the white noise + /s/ segments created for insertion into the “Added” condition, as described above. The speech sound mixed with noise constituted the “Added” stimuli. While white noise alone made up the “Replaced” stimuli.

### Paradigm

A two-alternative forced choice task was used (refer to Table 3 for *d*′, Beta and Miss Rate). In the word and pseudoword conditions, subjects heard a single stimulus at a time. Subjects were instructed for each token to determine if noise was replacing part of the word, “Replaced condition,” or if the noise coincided with part of word, “Added” condition (Samuel, 1981a). The word and pseudoword conditions occurred during the same block, and were pseudorandomized to prevent the added and replaced condition of individual stimuli from occurring sequentially. Subjects were not explicitly told that the paradigm consisted or words and pseudowords, nor that medial /s/ was the target phoneme. The speech sound segment condition always followed the word/pseudoword block. In the speech sound segment condition, subjects were asked to determine if they were hearing noise by itself or noise mixed with a speech sound (Samuel, 1981a). Subjects were not told that the speech sound was an /s/. A single block of speech sound segments followed the word and pseudoword block for every subject. Participants practiced the task with feedback in a randomized block consisting of 10 words and 10 pseudowords. Prior to the speech sound segment test block, participants practiced 10 speech sound segment trials. In all trials subjects had 4 s to answer per item, after which the trial timed out. These time-outs constituted no more than five trials within each block. No subjects were excluded based on missed trials.

### Statistical analysis

Following other studies of phonemic restoration (Sherman, 1971; Warren and Obusek, 1971; Warren and Sherman, 1974; Samuel, 1981a,b; Samuel and Ressler, 1986; Samuel, 1987, 1991), *d*′ was used as sensitivity measure of restoration (Macmillan and Creelman, 2004). In this experiment, *d*′ was calculated using the following formula: *d*′ = *z*(*H*)−*z*(*F*). Where “*H*” describes the hit rate (that is, proportion of “Noise Coincided” responses for the “Added” condition) and “*F*” describes the number of false alarms (that is, proportion of erroneous “Noise Coincided” responses for the “Replaced” condition) (refer to Table 3 for *d*′, Beta and Miss Rate).

First, paired *t*-test were used to confirm that our experiment showed the same pattern of restoration that has been previously reported (Samuel, 1981a); subjects were found to have stronger phonemic restoration for words than pseudowords, and greater restoration for both words and pseudowords compared to speech sound segments. Second, a correlation analysis was performed to determine if a relationship existed between reading ability and phonemic restoration and the magnitude of that relationship. Last, a multivariate analysis of variance (MANOVA) statistic was chosen to allow for the examination of the interaction between reading groups (good and poor-to-dyslexic readers) and all levels of restoration (word, pseudoword and speech sound segment conditions) while avoiding increasing the risk of an inflated Type I error. Preliminary statistical analysis confirmed a relationship between restoration conditions, but also demonstrated that the magnitude of these relationships was not uniform thus making the results ideally suited for MANOVA analysis (see Keppel and Wickens, 2004; Huberty and Olejnik, 2006).

## Results

It is worth reiterating that a low *d*′ score indicates that the two versions of stimuli, added and replaced, are not discriminable; the stimuli are perceived as alike because the missing phoneme signal is being restored in the replaced version. A low *d*′ score indicates high restoration. A high *d*′ score indicates that the critical segments do not sound alike and restoration is not occurring. Three paired samples *t*-tests were used to confirm the previously found pattern of restoration (Samuel, 1981a). A significant difference was found between word [condition mean (M) = 0.613, *SE* = 0.037] and pseudoword (*M* = 1.025, *SE* = 0.049) restoration *t*_(52)_ = −7.46, *p* = 9.0555^−10^. This indicates stronger restoration for words then pseudowords. A significant difference was found between word (*M* = 0.613, *SE* = 0.037) and speech sound segment (*M* = 2.160, *SE* = 0.108) restoration *t*_(52)_ = −14.322, *p* = 4.189^−13^. A significant difference was also found between pseudoword (*M* = 1.025, *SE* = 0.049) and speech sound segment (*M* = 2.160, *SE* = 0.108) restoration *t*_(52)_ = −9.599, *p* = 1.101^−19^. This indicates stronger restoration for both words and pseudowords compared to speech sound segments (see Table 3, bottom row). All comparisons were significant according to Bonferroni correction.

### Relationship of phonemic restoration to reading ability

**Figure 1:**
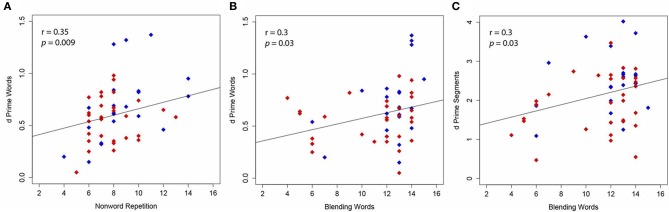
**Blue indicates good readers. Red indicates poor-to-dyslexic readers**. Correlations show the relationship between phonemic restoration and subtests of the Comprehensive Test of Phonological Processing (CTOPP). *A* high *d*′ score (y-axis) indicates that the critical segments are not perceived as alike and restoration does not occur. As such, the positive correlation reflects that better performance on the CTOPP subtest is associated with less restoration. Significant positive correlations were found between **(A)** Non-word Repetition subtest and word restoration **(B)** Blending Words subtest and word restoration **(C)** Blending Words subtest and speech sound segment restoration.

In Samuel (1981a), a single ecologically valid sample was used in which no reading assessments were used to distinguish between subjects. In the current study, participants were intentionally recruited across a range of reading ability based on standardized reading assessment scores. These scores indicate that our subject sample ranged from good-to-dyslexic readers (see Table 1). While there are many papers positing subtypes of dyslexia, here we aimed only to use dyslexic participants with a phonological awareness deficit. Individuals with additional/exclusive rapid naming deficits were excluded based on assessment scores (Wolf and Denckla, 2005). We ran a Pearson correlation in order to determine if a relationship existed between phonemic restoration and skills commonly associated with reading ability (see Table 1 for all nine subtests that were run for possible correlations). False discovery rate (FDR) was used to correct for multiple comparisons. Among these subtests, there was a positive correlation between *d*′ scores in the words condition and the standardized assessment scores on subtests of the CTOPP Blending Words *r* = 0.3, *p* = 0.03, and Non-word Repetition *r* = 0.35, *p* = 0.009 (see Figures 1A,B). Each of these subtests is used to assess phonological processing based on two components: phonological awareness and phonological memory. Given that high *d*′ scores indicate less restoration, this result shows that *better* performance on standardized assessments of phonological processing is correlated with *less* phoneme restoration in the word condition. There were no correlations between the restoration effect for pseudowords and any of the behavioral standardized reading assessments. There was a positive correlation between *d*′ for speech sound segments and the standardized assessment score for CTOPP Blending Words *r* = 0.3, *p* = 0.03, (see Figure 1C) indicating that better performance on this phonological processing subtest correlated with less restoration within the speech sound segment condition. Taken together this correlation seems to suggest that better readers, that is, those with high phonological processing skills, are less likely to show the restoration effects in words and speech sound segments.

### Difference between good readers and poor-to-dyslexic readers

Only one particular aspect of reading, phonological processing, appears to be correlated with phonemic restoration (at the word and speech sound segment condition levels). Given our preliminary findings (see Table 3) suggesting no differences between poor readers and dyslexic readers on measures on phonemic restoration, for the purposes of group analysis we will only compare good readers (*n* = 23; male = 8) and poor-to-dyslexic readers (*n* = 30; male = 10). While lexical representations enable native listeners to restore phonemes within real words, the pseudoword and speech sound segment conditions must rely more heavily on bottom-up acoustic information. We predict those with lower phonological awareness may present with additional difficulties in restoring pseudowords and speech sound segments.

**Figure 2:**
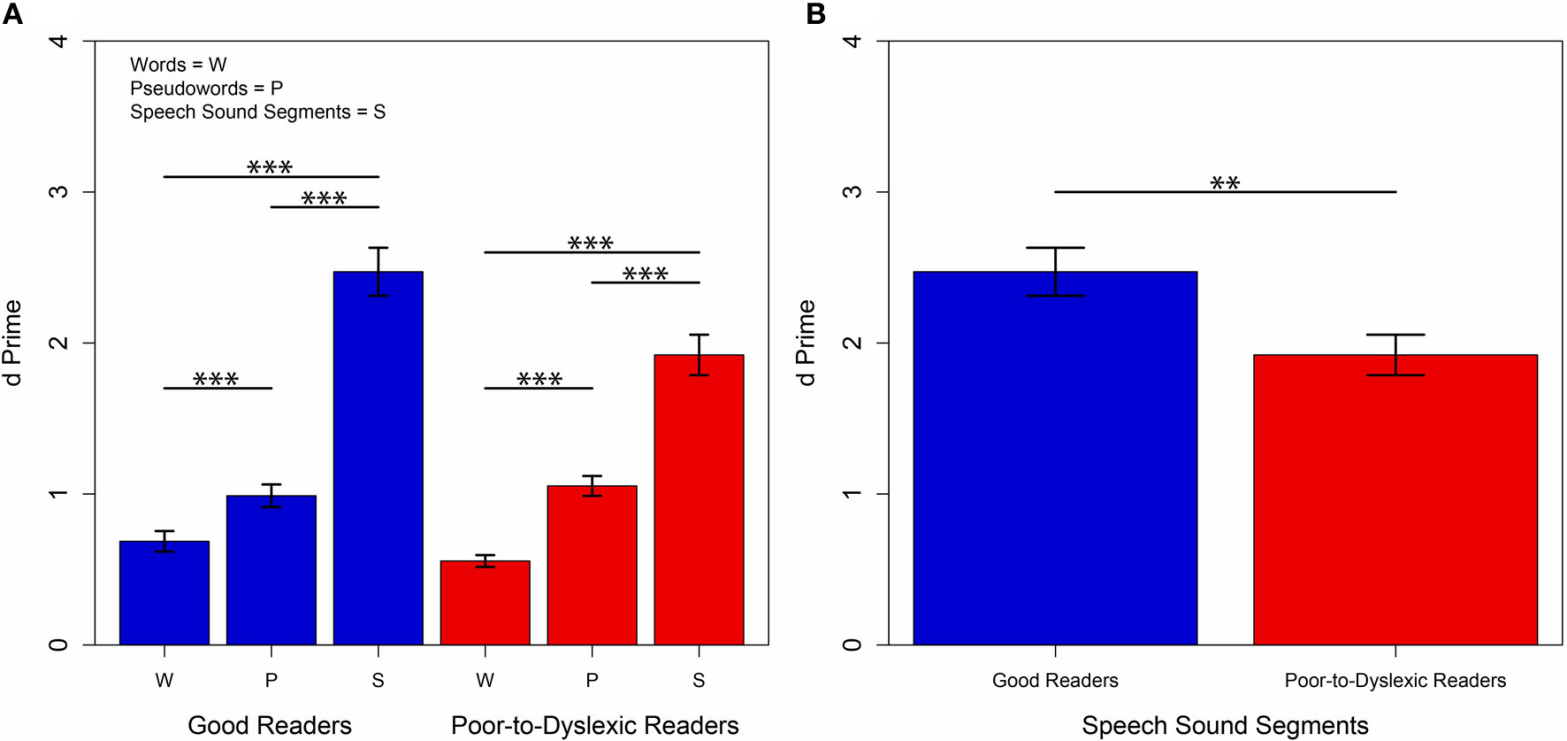
**Mean phonemic restoration performance by reading group: good readers and poor-to-dyslexic readers**. Low *d*′ scores indicate more susceptibility to the restoration illusion. **(A)** Amount of restoration is shown as a measure of *d*′ for good readers and poor-to-dyslexic readers across the word (W), pseudoword (P) and speech sound segment (S) conditions. Stronger restoration was found for words compared to pseudowords, words compared to speech sound segments and pseudowords compared to speech sound segments. **(B)** A main effect of group within the segments condition was found. Using a *post-hoc* Bonferroni adjusted alpha, a significant difference was found between good and poor-to-dyslexic readers ability to restore speech sound segments at *p* = 0.01. Error bars indicate standard error of the mean (s.e.m.). Asterisks indicate ^*^p < 0.05, ^**^p < 0.01, ^***^p < 0.001.

A MANOVA was conducted, with the three *d*′ measures of restoration (words, pseudowords and speech sound segments) as dependent variables, and reading group membership (good readers and poor-to-dyslexic readers) as the independent variable. Assumptions of homogeneity of the variance-covariance matrices as well as the assumption of equality of variance were met. A statistically significant difference found between good readers and poor-to-dyslexic readers on the combined measures of *d*′ restoration (2 Reading Groups^*^3 Condition Levels of Restoration) *F*_(3, 49)_ = 3.392, *p* = 0.025; Pillai's Trace = 0.172; partial eta squared = 0.172. Within groups, differences were again found between individual measures of restoration. Within groups (good readers and poor-to-dyslexic readers) differences on individual measures of restoration (words, pseudowords and speech sound segments) were investigated using a Bonferroni adjusted alpha level of 0.017. A significant difference was found for restoration of words, pseudowords and speech sound segments *F*_(3, 49)_ = 290.873 *p* = 3.315^−31^; Pillai's Trace = 0.947; partial eta squared = 0.947 (see Figure 2A). This again replicates previous results from Samuel (1981a) on an ecological sample. Here we again show that greater restoration is found for words (*M* = 0.613, *SE* = 0.037) than pseudowords (*M* = 1.025, *SE* = 0.049), and greater restoration for both words and pseudowords compared to speech sound segments (*M* = 2.160, *SE* = 0.108).

Between groups (good readers and poor-to-dyslexic readers) differences on individual measures of restoration (words, pseudowords and speech sound segments) were investigated using a Bonferroni adjusted alpha level of 0.017. No significant effect of group was found within the words and pseudowords conditions, suggesting that the group by condition interaction is largely driven by the performance of the good and dyslexic readers on the speech sound segment condition. The only measure of restoration to reach significance was the restoration of speech sound segments *F*_(1, 52)_ = 7.074, *p* = 0.010, partial eta squared = 0.122. Given that the assumption of equality of variance was met, a Bonferroni adjusted alpha of 0.025 was used for pairwise group mean comparison. A significant difference in speech sound segment restoration *p* = 0.010 was found, with a mean increase (*M* = 0.551, *SE* = 0.207, 95% CI: 0.135 and 0.967) in speech sound segment restoration for good readers (*M* = 2.472, *SE* = 0.159) compared with poor-to-dyslexic readers (*M* = 1.921, *SE* = 0.134) (see Figure 2B). The increase in good reader *d*′ for speech sound segment restoration indicates that good readers are less susceptible to the speech sound segment restoration illusion.

## Discussion

Across a diverse population that included good readers, poor readers, and individuals with dyslexia, restoration was found to occur *less* in those who show high measures of phonological processing on standardized assessments. That is to say, individuals with better phonological abilities may have greater reliability in their low-level acoustic-phonetic representations for these sounds, and are less “fooled” by the substitution of white noise for speech sounds. Across a continuum of abilities, individuals with less intact phonological abilities may rely more on lexical-semantic information, as suggested by prior evidence from studies of individuals with phonological deficits in dyslexia (Frith and Snowling, 1983). This pattern suggests that the acoustic-phonetic processing or retention of acoustic-phonetic detail is not as reliable in both poor and dyslexic readers.

Although the current findings suggest that there may be subtle deficits at the *single* segmental level in poor readers and individuals with dyslexia, it is noteworthy the types of tasks that are used to characterize the poor reading and dyslexic population are tasks that require subjects to *combine* sounds in childhood at the level of phonological processing and as adults at the whole word or pseudoword level. In a longitudinal study that began with training of individual speech sounds in kindergarteners at risk for reading failure, trained students outperformed their at-risk untrained peers by seventh grade; however, trained at-risk children were still grades below an untrained control group of typical readers (Elbro and Petersen, 2004). This begs the question: are phonological awareness impairments a deficit in phonological processing, which includes manipulating, subtracting and combining speech sounds or are phonological awareness impairments a symptom of a lower level phonetic failure? We offer two explanations of why poor phonological awareness could lead to differences in phonemic restoration.

### Perceptual stability deficit: account of dyslexic impairment

A first possibility is that those with more difficulty in phonological awareness show a failure of perceptual tuning stability. The ability to resolve fine-grained acoustic details relies upon having intact, stable representations of speech sounds. In particular, it may be the case that the “tuning curves” for phonetic categories for those with poor phonological awareness are shallower and wider than in the typical population. Unstable representations of speech at the individual sound level would result in poor speech sound boundaries, which may result in non-speech sounds (noise) being mistaken for speech sounds. Without a stable representation of speech at the individual speech sound level, those with phonological awareness deficits or lower phonological awareness ability may already be taxing their perceptual system to map incoming speech sounds into distinct categories, even without the interference of noise.

Phonemic restoration is thought to tap into important systems for typical adult speech perception. Specifically, phonemic restoration has been suggested to be a byproduct of the intact adult perceptual systems ability to handle mispronunciation, talker variability and accented speech (Frisch and Wright, 2002). Phonemic restoration has been shown to be persistent enough in the intact adult perception system that it can produce perceptual effects generally thought to be uniquely associated with “real” phonemes. In a selective adaptation design, along a voice-onset-time (VOT) continuum, subjects showed a continuum shift for restored phonemes similar to that seen for real phonemes (Samuel, 1997). Restoration has also been shown to be robust enough to compensate for co-articulation (Elman and McClelland, 1988) and to shift perception of vowel quality (Ohala and Feder, 1994). Evidence from imaging studies further suggests that restoration results in “real” sounds. A functional Magnetic Resonance Imaging (fMRI) study of tone restoration showed activation in Heschl's gyrus for both real tones as well as perceived continuity of imagined tones in noise (Riecke et al., 2007). Furthermore, extracellular recordings from macaque monkeys found that illusory tones in noise, like real tones in noise, elicit A1 single-neuron response in the primary auditory cortex (Petkov et al., 2003). Given previous evidence one could suggest that restoration is persistent enough to act as a replacement for auditory stimuli.

Shahin et al. (2009) suggested that anatomical areas activated for the restoration (illusion) which included Heschl's gyrus, the left posterior angular gyrus (AG), bilateral superior temporal sulcus (STS), and superior frontal sulcus (SFS) are distinct from regions specific to repair in restoration (illusion > illusion failure), which included Broca's area, the pars opercularis, and bilateral anterior insula. Only unconscious restoration illusion is robust enough to elicit the same neuronal pathway that is activated for natural speech perception, while noted degradation (e.g., repair in restoration: illusion > illusion failure) to the stimuli being restored elicits a different neuronal network. As in the Shahin study, participants in the present study showed a mix of responses such that sometimes they did not detect the absence of the phoneme (illusion) and sometimes did detect this absence (illusion failure). The repair network that is utilized in the restoration of speech, the network recognized by the listener as degraded (speech mixed with noise), is made up of a network utilized in the acquisition of unfamiliar auditory inputs (Myers and Swan, 2012) and in auditory expertise (Zatorre et al., 1992). Taken together this further suggests that that phonemic restoration is tapping into the ability of the perceptual system to handle variability (Frisch and Wright, 2002). As such, we speculate that over-restoration of individual speech-segments could potentially impact speech perception in the real world, not simply the lab.

Current results are consistent with the suggestion that deficits in phonological awareness seen primarily in individuals with dyslexia are at least in part a consequence of a failure at the phonetic level (Morais et al., 1987). Additionally (or alternatively), deficits at the phonetic level may be due to a weaker top-down compensatory mechanisms (Boets et al., 2013). Speech perception requires that listeners map the complex acoustic (bottom-up) signal of speech onto speech sound (i.e., phonetic) categories. To accomplish this, the listener must perceive fine-grained details of the acoustic (bottom-up) signal in order to extract the temporal and spectral information and connect that information to a known phonetic category using top-down information. The categorical perception paradigm allows for the examination of phonetic perception stability. The acoustic differences within a speech category (e.g., two different examples of /d/) are difficult to distinguish, whereas acoustic differences that result in a change in category (e.g., a /d/ and a /b/) are very easy to distinguish (Liberman et al., 1957). Categorical perception has been explored in children with reading difficulty in French (Joanisse et al., 2000), Chinese (Liu et al., 2009), Dutch (Maassen et al., 2001), and English (Werker and Tees, 1987). Results from studies of categorical perception in children report mixed findings. Although some studies report less-categorical perception in dyslexic children (Werker and Tees, 1987; De Weirdt, 1988; Maassen et al., 2001; Bogliotti et al., 2008; Liu et al., 2009), studies have also shown dyslexic children with normal performance on categorical perception (Brandt and Rosen, 1980; Manis et al., 1997; Joanisse et al., 2000). These divergent findings may result from differences in criteria for dyslexia used in the study. Noordenbos et al. (2012) provided initial evidence suggesting that differences in categorical perception in dyslexic children may increase with age, such that as children mature, they become even more different from their peers. Such a maturational effect may partially explain the contradictory results reported above, due to age variance across studies. It is worth reiterating that, as a group, individuals with dyslexia performed similar to controls on restoration in the context of words and pseudowords, although individual differences in restoration across the population were correlated with standardized measures of phonological processing. This finding may reflect preserved access to top-down information from lexical status. In an adult study of categorical perception, Ruff et al. (2003) showed that French dyslexic individuals did not show typical patterns of brain activation for phonetic changes in stimuli (between category > within category) in the left angular gyrus, right inferior frontal gyrus, and the right cingulum. This is consistent with evidence that dyslexic children are able to capitalize on top-down lexical status to perform categorical perception tasks (Reed, 1989; Serniclaes et al., 2001, 2004; Bogliotti et al., 2008).

### Inappropriate perceptual auditory plasticity: a second account of dyslexic impairment

A second or additional possibility is that those with phonological difficulty show inappropriate auditory plasticity. Degraded or impaired phonological processing may reflect a neural architecture that is overly plastic and, as a consequence shows so much accommodation of new sounds that non-speech sounds are inappropriately assimilated into the phonetic category. Prior evidence also suggests that when phonetic units of speech are acoustically exaggerated, infants (Liu et al., 2003) and children with dyslexia (Tallal et al., 1996) are better able to demonstrate phonetic perception. Evidence from studies of perceptual learning paradigms in both animals and humans seems to suggest that perceptual learning is associated with a high degree of experience-dependent plasticity (Sisneros et al., 2004; Golestani et al., 2011; Stein et al., 2012). We speculate that individuals with dyslexia may be showing delayed maturation effects; dyslexic individuals may be unable to exit a critical period in which no “neuronal commitment” has been made to their native language (see Kuhl and Rivera-Gaxiola, 2008). Therefore, an inability to separate speech and noise in a phonetically impaired system may be the result of a deficit in separating noise from a phonetically plausible acoustic signal. A speech system that has remained plastic for prolonged period of time, without a commitment to distinctive speech sound categories, may be willing to accept white noise as a new speech sound.

In this paper we provide evidence that a phonetic impairment does exist in dyslexia, in particular that single speech sounds embedded in noise are likely to be confused with the background noise. At the same time, we show that individuals with dyslexia are able to capitalize on lexical knowledge and phonotactic information to overcome hypothesized perceptual difficulties at the speech sound segment level. Recent evidence suggests that phonetic information is processed in a typical way in the STG but less accessible in individuals with dyslexia due to a degraded connection between the STG and the IFG (Boets et al., 2013). In contrast, our results indicate that, at least for phoneme restoration, the use of top-down information from the lexicon is indistinguishable from that seen in typical adults. Future remediation studies in dyslexia should focus on strengthening acoustic representations at the phonetic level, while also strengthening top-down connection through increasing the amount of perceptual noise while training children on basic reading skills.

### Conflict of interest statement

The authors declare that the research was conducted in the absence of any commercial or financial relationships that could be construed as a potential conflict of interest.
